# Rise of mucormycosis during the COVID-19 pandemic and the challenges faced

**DOI:** 10.18502/cmm.2023.345032.1400

**Published:** 2023-03

**Authors:** Malavika Kottarathil, Premamalini Thayanidhi, Sathyamurthy P, Anupma Jyoti Kindo

**Affiliations:** 1 Department of Microbiology, Sri Ramachandra Institute of Higher Education and Research, Chennai, India; 2 Department of General Medicine, Sri Ramachandra Institute of Higher Education and Research, Chennai, India

**Keywords:** COVID-19, Molecular diagnosis, Mucormycosis, Zygomycosis

## Abstract

Mucormycosis (previously called zygomycosis) is a diverse group of increasingly recognized and frequently fatal mycotic diseases caused by members of the class zygomycetes. Mucormycosis is around 80 times more common in India, compared to other developed countries, with a frequency of 0.14 cases per 1,000 population. The most frequent causative agent of mucormycosis is the following genera from the Order *Mucorales Rhizopus*, *Mucor*, *Rhizomucor*, *Absidia*, *Apophysomyces*, *Cunninghamella*, and *Saksenaea*. The major risk factors for the development of mucormycosis are diabetic ketoacidosis, deferoxamine treatment, cancer, solid organ or bone marrow transplantations, prolonged steroid use, extreme malnutrition, and neutropenia. The common clinical forms of mucormycosis are rhino-orbital-cerebral, pulmonary, cutaneous, and gastrointestinal. During the second wave of COVID-19, there was a rapid increase in mucormycosis with more severity than before. Amphotericin B is currently found to be an effective drug as it is found to have a broad-spectrum activity and posaconazole is used as a salvage therapy. Newer triazole isavuconazole is also found effective against mucormycosis. This study aimed to review various studies on the laboratory diagnosis and treatment of mucormycosis

## Introduction

The class Zygomycetes includes two orders: Mucorales and Entomophthorales. Common genera that cause mucormycosis, like *Mucor*, *Rhizopus*, *Rhizomucor*, *Absidia*, *Apophysomyces*, *Cunninghamella*, and *Saksenaea* are under the order Mucorales while order Entomophthorales includes *Conidiobolus* sp. and *Basidiobolus* sp. that cause cutaneous and subcutaneous infections [ 1 ]. Their prevalence rates in India are around 80 times more than the other developed countries [ 2 ]. Zygomycetes are associated with disseminated and frequently fatal infections due to their angio-invasive nature causing tissue infarction, especially in immunocompromised individuals. 

The risk factors commonly encountered are diabetic ketoacidosis, deferoxamine treatment, cancer, solid organ or bone marrow transplantations, prolonged usage of steroids, extreme malnutrition, neutropenia, trauma, burns, or neonatal prematurity [ 3 - 5 ]. Few studies have reported that the use of immunosuppressants, like azathioprine, had increased the risk of mucormycosis in patients with inflammatory bowel disease, making it another risk factor [ 6 - 8 ].

The diagnosis of mucormycosis is difficult, and the main challenge is the identification of zygomycetes to the species level isolated in culture and the direct identification in tissue specimens. However, there have been recent advances in new molecular approaches. Delay in the accurate identification of zygomycete infections can negatively affect patient survival [ 9 ]. Timely diagnosis of invasive mucormycosis is important for improved survival rate, early treatment, and reduced morbidity as it is one of the most fulminant mycoses. 

During the COVID-19 pandemic, a remarkable increase in the incidence rate of mucormycosis was noted in India. Moreover, zygomycetes show resistance against conventional antifungal agents; therefore, there is a need to study newer antifungal agents for the treatment [ 10 ]. During the second wave of the COVID-19 pandemic, which raised the mortality and morbidity rates, mucormycosis gained significance. Recently, documented incidences of mucormycosis have increased during the SARS-CoV-2 pandemic, leading to serious illnesses in COVID-19 patients. The management of COVID-19 disease has become more challenging by the rising prevalence of COVID-19-associated mucormycosis (CAM). During the COVID-19 pandemic, such invasive fungal diseases are on the rise and a cause for concern [ 11 ]. This study aimed to highlight the rise of mucormycosis during COVID-19 and the challenges in its diagnosis. It also aimed to shed light on the differences observed in this regard between the pre-COVID-19 and COVID-19 periods.

## Materials and Methods

The electronic databases of PubMed, Scopus, and Google Scholar were searched for a structured review of the literature up until January 2023 using the terms "Mucormycosis," "COVID-19," "Zygomycosis," "SARS CoV-2," "*Mucor*," and "*Rhizopus*," as well as "CAM risk factors," "Clinical complications in CAM," and "Diagnosis of Mucormycosis and CAM.". The corresponding authors have independently verified the accuracy of the data. A total of 128 works of literature were included in this study.

### Epidemiology: then and now

Globally, the incidence rate of mucormycosis varies from 0.005 to 1.7 per a million population. The prevalence rate of India is estimated to be 140 per million population, which is about 80 times higher than that of other developed countries [ 12 ]. A systemic review and meta-analysis of 851 case reports published in 2018 has reported death in 389 out of 851 (46%) patients. Case fatality was observed to be highest among patients with disseminated mucormycosis (68%) and lowest in those with cutaneous disease (31%). 

Based on a study performed by Jeong et al., the cases of mucormycosis reported in Europe were higher, compared to Asia. However, results of a recent review of 851 cases reported over a period from 2000 to 2017 were different as the rate of mucormycosis cases was 34% in Europe, 31% in Asia, 28% in North or South America, 3% in Africa followed by 3% in Australia and New Zealand [ 13 ]. The highest number of cases are encountered in India but due to the lack of population-based studies, the exact incidence rate is unknown. 

In India and throughout the world, the leading cause of mucormycosis is *Rhizopus arrhizus*. The variety of agents causing mucormycosis in India is considerably wide. The second most frequently isolated agent is *Apophysomyces variabilis*. Based on the literature, *Apophysomyces* species cause necrotizing fasciitis i.e., cutaneous mucormycosis, and are responsible for about 60% of reported cases. 

Species of *Lichtheimia* account for 0.5-13% of cases in India. According to Chander et al., *L. ramosa* is the cause of the majority of cases in India. Other genera associated with mucormycosis in India are *Rhizomucor pusillus*, *Mucor* species *Cunninghamella species*, *Syncephalastrum* species, and *Saksenaea* species [ 14 ].

Mucormycosis is also reported due to rarer organisms, like *Saksenaea erythrospora*, *Mucor irregularis*, and *Thamnostylum lucknowense* [ 15 ]. Its incidence rate was estimated to have increased 2.1 times during the COVID-19 outbreak, with a sharp increase in cases of mucormycosis in patients with COVID-19, notably those from India [ 16 ]. During the SARS-CoV-2 pandemic, both developed and developing nations continue to bear the burden of severe secondary fungal infections. Mucormycosis cases are more common in underdeveloped countries, compared to developed nations, and the greatest number of CAM cases (incidence rate: 0.14 per 1,000 people) have been documented in India [ 17 ]. 

Hussain et al. carried out a meta-analysis on a sample size of 52,196 COVID-19 patients to estimate the global prevalence of CAM. They observed that the CAM incidence rate was 50 times greater (7 cases per 1000 COVID-19 patients) than the highest-incidence rates reported in previously published statistics (0.14 cases per 1,000 population). Infection with CAM was shown to have a significant death rate (26.9%) [ 18 ]. 

Nagalli et al. used electronically accessible data to conduct a systematic review. They identified 115 COVID-19 cases who had confirmed infection with mucormycosis. They stated that diabetes mellitus (DM) was the most prevalent co-morbidity for mucormycosis among them (approximately 77.1%) and that 90% of patients took steroids for the treatment of COVID-19. Antifungal therapy was used; however, CAM infection still had a significant death rate (approximately 48.7%) [ 19 ]. According to a study performed by Kamat et al., the majority of CAM cases were involved with rhino-orbital mucormycosis. More than 80% of individuals had a history of taking steroids and were diabetic. Despite the use of antifungal medication therapy, a mortality rate of 25.6% was recorded [ 20 ]. 

### Risk Factors

Diabetic ketoacidosis, deferoxamine treatment, cancer, solid organ or bone marrow transplantations, prolonged steroid use, extreme malnutrition, and neutropenia are the major risk factors for mucormycosis [ 3 ]. In Indian patients, trauma was also reported as a common risk factor [ 14 ]. Pulmonary mucormycosis is commonly reported in solid organ transplant recipients, and hematological malignancy, DM [ 15 ], and uncontrolled DM patients, especially those with ketoacidosis, are at a higher risk. In a multi-centric study performed in India, 465 cases were reported from 12 centers over 21 months. A study conducted by Patel et al. reported that there was an annual incidence rate of 22 cases per year and an average of 38.8 cases for each participating center [ 16 ]. 

A prevalence of 0.16-1.72% mucormycosis cases from North India was reported in patients with DM [ 21 , 22 ]. Prakash et al. also reported a higher risk of developing mucormycosis in North India (67%), compared to South India (22%) due to the higher rates of DM [ 23 ]. However, according to the results of studies carried out by Manesh et al., and Priya et al., there was no such regional difference in recent case series in South India (65.2-76.3%) [ 24 , 25 ], North India (54-62.2%), and Western India (55.6%). 

Diabetes mellitus is a major risk factor for mucormycosis with 72% cases in Mexico, 75% in Iran, and 52% in the USA, similar to India. In comparison, European countries have a lower prevalence of diabetes among mucormycosis cases (17-23%) [ 15 ]. Patients undergoing kidney dialysis are also at a higher risk due to deferoxamine therapy [ 26 , 27 ]. Moreover, excessive usage of steroids has been identified as a second major risk factor for mucormycosis [ 28 ]. Cirrhosis, carcinoma, anaemia, congenital heart disease, malnutrition, hepatitis, glomerulonephritis, uremia, amoebiasis, typhoid fever, and gastroenteritis have also been associated with mucormycosis [ 1 ]. All of them are common risk factors seen in both CAM as well as non-CAM infections; however, newly detected DM was an important risk factor in CAM [ 16 ]. 

### COVID-19 associated mucormycosis

In light of the rise in mucormycosis cases stimulated by the COVID-19 pandemic, the entity has been termed CAM [ 29 ]. Diabetes mellitus was detected for the first time in 20% of CAM cases during the COVID-19 pandemic as it has been shown that SARS-CoV-2 affects the pancreatic beta cells, leading to metabolic abnormalities that may result in diabetes [ 16 , 30 , 31 ]. The CAM is an emerging threat that necessitates increased attention to COVID‐19 patients, even those who have recovered as it portends a poor prognosis and demands an early diagnosis and treatment [ 29 ]. 

The CAM may occur due to COVID-19-induced immune dysregulation or associated treatments with corticosteroids and immunomodulatory drugs (e.g., tocilizumab or baricitinib) which impair the host defense mechanism against molds [ 32 ]. Owing to the unavailability of hospital beds during the second wave of the COVID-19 pandemic, many patients received additional oxygen via an oxygen concentrator in their homes. This led to an increased risk of developing mucormycosis infections due to unsanitary conditions [ 33 ]. 

The increase in mucormycosis cases in India could also be associated with high ferritin levels. In severe COVID-19 cases, there was a change in iron metabolism [ 34 ]. A study performed by Arora et al. in 2022 reported that CAM occurs soon after recovery from the COVID-19 infection. Moreover, in the aforementioned study, it was shown that there might be a link between novel non-conventional risk factors in CAM infection. It should also be noted that its prevalence was higher in rural areas, compared to urban areas, which could be due to a lack of proper treatment [ 35 ].

Steroids are frequently used for the treatment of pneumonia due to COVID-19. They lessen phagocytic activity which results in pathogen invasion that worsens glycemic control. This is challenging particularly when patients need steroids for the management of severe acute respiratory distress syndrome and COVID-19 pneumonia [ 36 ]. In a study carried out by Madhavan et al., it was reported that immunocompromised individuals receiving a higher dose of prednisone are at a higher risk of developing CAM [ 37 ]. Usage of glucocorticoids reduces the mortality rate in hypoxemic patients with COVID-19, however, it can increase the risk of developing a secondary infection [ 38 ]. The triad of SARS-CoV2, uncontrolled diabetes, and steroids is the sole reason for the increased incidence of mucormycosis [ 39 ]

Physical factors, like frequent nasopharyngeal swab testing (more than twice), for COVID-19, have been an independent factor associated with a higher risk of CAM. It may also induce micro-trauma to the nasal and nasopharyngeal mucosa, leading to angioinvasion. Cutaneous mucormycosis can occur at sites of local trauma, such as contaminated intravenous catheters, bandages, and needles. Similarly, it has been proposed that inhaling steam can lead to thermal damage to the nasal mucosa which can also be one of the risk factors [ 13 , 35 , 40 ]. The immunocompromised or immune-competent state of the individuals can be a predisposing factor for mucormycosis. 

Indirect factors, including interventions associated with the prevention and management of COVID-19, can also be a reason for the increased number of cases during the pandemic [ 35 ]. During the second wave, the COVID-19 pandemic has also potentially influenced a higher number of cases of mucormycosis, thus becoming an important risk factor. Prolonged stay in the intensive care unit and pulmonary and brain involvement with Mucorales also have a direct influence on increasing the risk of mortality [ 16 ]. However, in a study performed by Arora et al. in 2022, it was reported that 2-32% of cases of CAM may lack any of these associations. They also reported a single case of CAM, where the individual was neither diabetic nor had received steroids [ 35 ]. Potentially promoting new-onset diabetes, COVID-19 creates the circumstances for the emergence of mucormycosis [ 41 ].

### Breakthrough mucormycosis with the usage of voriconazole

The term "breakthrough invasive fungal infection" (bIFI) refers to an infection that develops after exposure to an antifungal agent which involves fungi that are not within the range of activity of antifungals [ 42 ]. Some of the major risk factors for bIFI include persistent neutropenia, immunosuppression, acute leukemia, and mucositis, which can either be a natural part of the underlying illness state or occur due to targeted immunosuppression or transplant. Other risk factors include the use of central venous catheters or antibiotics, which are frequently required to administer nourishment, fluids, or medication to prevent bacterial infections in the post-transplant phase or in the intensive care unit, or in the case of antibiotics. 

An aspect that contributes to the development of breakthrough invasive candidiasis (bIC) is exposure to two or more antibiotics for at least 14 days. This suggests changes in the endogenous microbiota (such as those of the skin and gastrointestinal tract) [ 43 ]. Few studies have reported that voriconazole (VRC) treatment for invasive aspergillosis or other non-*Aspergillus* mold invasive illnesses is associated with breakthrough mucormycosis [ 44 ]. According to a study, individuals with hematologic malignancies, who had previously taken VRC, had a higher risk of developing mucormycosis, with fatality rates ranging from 44.4 to 73% [ 45 ]. 

A retrospective study performed by Kim et al. reported the prevalence and incidence rates of breakthrough IFDs during VRC treatment for IA to be 2.25% and 0.22 cases per year, respectively [ 46 ]. Two prospective studies that investigated the association between VRC and breakthrough mucormycosis (Transplant Associated Infection Surveillance Network study of 393 transplant recipients and the prospective survey of invasive fungal infections) found that VRC was more frequently linked to breakthrough infections caused by zygomycetes and *Fusarium* spp. than *Aspergillus* spp. [ 47 ]. 

### Pathogenesis and clinical manifestations of mucormycosis

Clinical forms of mucormycosis are classified according to the anatomical site of infection which is as follows: rhino-cerebral or rhino-orbital, pulmonary, cutaneous, gastrointestinal, disseminated, or other uncommon presentations, like endocarditis, osteomyelitis, peritonitis, and renal infection [ 48 ]. The pathogenetic hallmark of the disease is the rapid and aggressive invasion of blood vessels, resulting in vessel thrombosis, tissue necrosis, and hematogenous dissemination of the fungus in all invasive clinical presentations. Factors associated with increased mortality rates include prolonged neutropenia, site of infection and disseminated disease, delayed treatment, infection due to *Cunninghamella* spp., and increasing age [ 13 , 49 , 50 ]. 

Pulmonary mucormycosis has non-specific clinical signs that make it difficult to distinguish it from pulmonary aspergillosis. Non-productive cough is a common symptom, whereas hemoptysis, pleuritic chest pain, and dyspnea are less common. Rarely, particularly in diabetics, pulmonary mucormycosis may present as an endobronchial or tracheal lesion. A summary of distribution, risk factors, and clinical forms of Mucormycosis is presented in Table 1.

**Table 1:** Summary of distribution, risk factors, and clinical forms of mucormycosis

Clinical forms	Percentage of distribution [ 50 - 58 , 62 ]	Mode of transmission [ 50 - 58 , 62 ]	Risk factors [ 15 , 50 - 58 , 62 ]	Clinical manifestations [ 50 - 58 , 62 ]	Mortality rate [ 15 , 50 - 58 , 62 ]	Region-wise prevalence [ 21 , 59 , 60 , 61 , 62 ]
Pulmonary mucormycosis	24%	Inhalation of sporangiospores	Neutropenic patients with cancer undergoing induction chemotherapy and patients undergoing HSCT with graft-versus-host disease	Non-productive cough, hemoptysis, pleuritic chest pain, dyspnea	76%	India and Europe (0.01-14/1,00,000 population), China (52.5%)
Rhinocerebral mucormycosis	48%	Inhalation of sporangiospores into paranasal sinuses	Common in patients with diabetes mellitus, underlying malignancies, recipients of hematopoietic stem cell or solid organ transplants, and individuals with other risk factors, like diabetes	Sinusitis and periorbital cellulitis, facial pain, facial numbness, blurry vision, multiple cranial nerve palsies, unilateral periorbital facial pain, orbital inflammation, eyelid edema, blepharoptosis, proptosis, acute ocular motility changes, internal or external ophthalmoplegia, headache, acute vision loss, and fever	40-50%	India (9,10,000/year), China (10,000/year), USA (3 cases/a million population), UK (0.9 cases/a million population), Canada (1.2 cases/a million population), Australia (0.6 cases/a million population)
Cutaneous mucormycosis	19%	Inoculation of fungal spores	Diabetic ketoacidosis, deferoxamine treatment, cancer, solid organ or bone marrow transplantations, prolonged steroid use, extreme malnutrition, and neutropenia	Necrotic eschar accompanied by surrounding erythema and induration, nonspecific erythematous macule	4-10%	North America (256 cases, 36.9%), Asia (216 cases,31.2%) Europe (149, 21.5%) Central and South America (29, 4.2%), Australia (n=40, 5.8%) Africa (n=3,0.4%)
Gastrointestinal mucormycosis	~7%	Ingestion of the fungal spores	Disease reported mainly in premature neonates, malnourished children, and individuals with hematological malignancies, diabetes mellitus, or a history of corticosteroid use	Bowel perforation, peritonitis, sepsis, and massive gastrointestinal hemorrhage	85%	Asia (n=89,50.6%) North America (n=33,18.75%) Africa (n=26,14.8%) Europe (n=16,9.1%) Australia (n=8,4.5%) South America (n=4,2.3%) India (n=67) USA (n=28) South Africa (n=24)
Disseminated mucormycosis	9%	Hematogeneous	Iron overload (especially those receiving deferoxamine), profound immunosuppression (e.g., recipients of allogeneic stem cell transplants having graft-versus-host disease treated with corticosteroids), or profound neutropenia and active leukemia	Vascular invasion and tissue infarction in the affected organs. A metastatic skin lesion is an important hallmark in early diagnosis	96%	India (0.5-9%) China (9.2%) Iran (n=8) Australia (39.2%) Middle East (19%) North Africa (19%)

### Other uncommon forms of mucormycosis

Other uncommon forms include endocarditis, osteomyelitis, peritonitis, and pyelonephritis. Endocarditis of prosthetic or native valves can occasionally be caused by mucormycosis. Aortic thrombosis can be caused by endocarditis, which develops on or near prosthetic valves. Osteomyelitis typically develops following a traumatic inoculation or surgical procedure (such as the implantation of a tibial pin or the repair of the anterior cruciate ligament). 

Hematogenous osteomyelitis is relatively uncommon, but researchers have described osteomyelitis of the tibia, cuboid, calcaneus, femur, humerus, scapula, metacarpals, and phalanges [ 63 - 69 ]. It is also uncommon for patients receiving continuous ambulatory peritoneal dialysis to have zygomycetes involved in the peritoneal cavity. Although the attributable death rate in patients receiving inadequate or delayed treatment has reached 60%, peritonitis often progresses slowly. Rarely, renal mucormycosis should be suspected in any immunocompromised patient presenting with hematuria, flank pain, and unexplained anuric renal failure. Another uncommon indication of mucormycosis is brain involvement (usually in the basal ganglia) in leukemia patients and intravenous drug users without rhino-orbital involvement. Mastoid, oral mucosa, bone, bladder, trachea, mediastinum, or ear mucormycosis that is isolated from other cases is uncommon [ 52 ].

### Clinical Complications in COVID-19 Associated Mucormycosis

Clinical forms of mucormycosis can be categorized into rhino-orbital-cerebral, pulmonary, cutaneous, gastrointestinal, and disseminated mucormycosis. During the COVID-19 pandemic, complications in clinical presentations were observed in CAM cases. A study conducted by Pruthi et al. described five cases of pulmonary mucormycosis linked to COVID-19 that were complicated by pulmonary artery pseudo-aneurysm. When combined with comparable radiographic findings in mechanically ventilated patients, the detection of a mucormycosis agent from respiratory specimens supports the diagnosis [ 70 ]. 

Facial pain, facial palsy, headache, loose teeth, black necrotic turbinate, peri-orbital or facial edema, skin induration, and blackish staining are some of the clinical signs of head and neck mucormycosis. Moreover, bloody nasal discharge and palate damage are symptoms associated with nasal and oral cavity invasion. As a result of orbital expansion, the ophthalmic artery, and optic nerves may be destroyed, which could result in eyelid ptosis, proptosis, visual problems, and blindness.

A retrospective study performed in India reported vision loss in patients with CAM [ 71 ]. Due to extension from the orbit, cavernous sinus involvement can cause diplopia and ophthalmoplegia. In addition, cerebral involvement was noted in patients with CAM. Meningitis, cavernous sinus thrombosis, fungal abscess, and cerebrovascular illness are just a few examples of how the brain is involved. Ten studies

presented information on the provision of invasive or non-invasive mechanical ventilation for patients with pulmonary mucormycosis linked with COVID-19. Fever, dyspnea, cough, chest discomfort, and hemoptysis are signs of pulmonary mucormycosis in non-ventilated individuals. Disseminated mucor-mycosis, which results from bloodstream invasion in highly immunocompromised patients, may damage any organ but primarily the brain and lungs, and causes non-specific symptoms, such as abdominal pain and distension, diarrhea, and gastrointestinal bleeding [ 71 ].

Most of the clinical presentations are similar in CAM and non-CAM infections; however, the percentage of complications encountered during the COVID-19 pandemic was higher which may be attributed to the increased invasiveness of zygomycetes during COVID infection. 

Rhinocerebral mucormycosis is the common clinical presentation seen in CAM, whereas pulmonary mucormycosis is reported in non-CAM infections. A study conducted by Watanabe et al. in 2022 reported that one-fifth of CAM patients may lose their eyes [ 72 ].

### Diagnosis

Diagnosis of mucormycosis is challenging as most of the cases are culture-negative and microscopic identification can sometimes miss the fungus due to improper sample processing or lack of expertise. Demand for a quick diagnosis of mucormycosis has been encountered during the COVID-19 pandemic due to its ability to cause infection. Various examinations for the identification of the pathogen are listed in Tables 2 and 3. 

**Table 2:** Various methods and their parameters in the diagnosis of mucormycosis

Samples	Diagnostic method	Turnaround time	Yield	Sensitivity	Specificity
Samples:	**Microscopic examination**	1-2 h	87.61%	89.17%	100%
	Potassium hydroxide wet mount [ 73 - 77 ]				
Tissue, blood/serum, bronchoalveolar lavage, bronchial wash, urine	Calcofluor white stain [ 77 ]	1-2 h	87.61%	89.17%	100%
	**Histopathological examination**	3-4 days	95%	81.5%	100%
	Formalin-fixed paraffin-embedded [ 77 ]				
	Fresh tissue [ 77 ]	3-4 days	100%	100%	100%
Samples:	Culture [ 75 - 77 ]	3-5 days	44.24%	34.39%	100%
Tissue, Blood/serum, Broncho alveolar lavage, Bronchial wash, Urine					
Culture	Molecular tests*:	≤24 h	100%	97.45%	97.56%
Samples:	Real-time PCR [ 76 ]				
Tissue, blood/serum, bronchoalveolar lavage, bronchial wash, urine	Panfungal PCR+sequencing [ 76 ]	≤24 h	100%	95.54%	82.93%
	Mucorales specific [ 78 ]	≤24 h	100%	92.9%	100%

PCR: polymerase chain reaction

* Molecular diagnosis of mucormycosis from direct samples elaborated in Table 3

**Table 3:** Molecular identification directly from clinical samples

Samples	Type of PCR	Target regions
FFPE tissue	Conventional PCR	ITS region
		Panfungal marker [ 79 ]
BAL, plasma, and lung tissue	Real-time PCR	28S rDNA [ 80 ]
Fresh and FFPE tissue	Real-time PCR	Cytochrome b gene [ 9 ]
Plasma, urine, and BAL	Conventional PCR	Cot H (spore coating gene) [ 81 ]
FFPE tissue and spiked human blood sample	Real-time PCR	Rnl mitochondrial gene [ 82 ]

PCR: polymerase chain reaction, FFPE: formalin fixed paraffin embedded, BAL: bronchoalveolar lavage, ITS: internal transcribed spacer

According to previous studies performed by Bonin et al. and Dannaoui et al., the sensitivity of testing fresh tissue was 100% while that of testing formalin-fixed and paraffin-embedded tissue was only 56%. Low sensitivity in formalin-fixed tissue was due to the nucleic acid fragmentation by the extensive cross-linkage of tissue proteins after fixation in formaldehyde which is known to inhibit the polymerase chain reaction (PCR) process and can also give false negative results [ 83 , 79 ]. Diagnosis of mucormycosis from direct clinical samples aids in rapid identification thus helping in preventing clinical complications and reducing the morbidity and mortality rates of COVID-19 patients.

### Serological Tests

The various serological tests used for the diagnosis of mucormycosis including Enzyme-linked immune-sorbent assay (ELISA) (*R. arrhizus* WSSA) [ 84 ], Western blot [ 84 ], ELISA (ELISpot) or Immunocytofluorimetric assay-IFN-γ-producing T cells specific for Mucorales [ 85 ], and ELISA (Protein antigen RSA of 23 kDa) [ 86 ]. These are not routinely performed and are yet to be validated for the diagnosis. 

### Molecular Methods

Pulse field gel electrophoresis is a useful tool for molecular typing and revealing the genetic variability at species and intraspecies levels. Karyotypes of several zygomycetes, like *Mucor circinelloides*, *M. bainieri*, *M. mucedo*, *M. plumbeus*, and *M. racemosus* [ 87 ], have been established. However, there are currently very few reports on opportunistic zygomycetes.

Randomly amplified polymorphic DNA analysis uses random oligonucleotide primers for the detection of distinctive amplification patterns. The intraspecies and interspecies genetic variability of *Rhizomucor* and *Rhizopus* species has also been studied by this method as they are among the clinically important zygomycetes [ 88 , 89 ]

Rolling circle amplification (RCA) is an isothermal amplification method proven to be rapid, cost-effective, and specific for the molecular identification of pathogenic fungi. They have a specific detection limit of a single nucleotide. In Mucorales, there was no cross-reactivity observed within tested strains. The RCA has rapidly identified the following six virulent species: *Rhizopus microspores*, *R. arrhizus*. *var. arrhizus*, *R. arrhizus* *var. delemar*, *Mucor irregularis*, *Mucor circinelloides*, *L. ramosa*, and *Lichtheimia corymbifera*. The RCA is particularly suitable for high-throughput applications. In addition, it is used as a screening tool in view of hospital hygiene and for understanding pathogen population dynamics. The development of a reliable diagnostic tool for the early detection and identification of fungi remains a priority for improving patient outcomes. Given the simplicity and the results, RCA can become a routine test in hospital hygiene when there is a huge number of samples to be screened [ 90 ].

### Automated Systems

Mass spectroscopy using MALDI-TOF is a good alternative to molecular methods in the setting of a microbiology laboratory due to its numerous benefits over other identification techniques, including ease of handling, cheap prices, speed, and the potential for high throughput. Filamentous fungi, like *Aspergillus* spp. as well as *Fusarium* sp., and to a lesser extent, the Mucorales have also been evaluated. The precision and simplicity of this identification method together with its cost-effectiveness and potential for high throughput, are its main advantages over molecular-based identification. The method does have limitations; accordingly, the isolates must be cultivated as pure cultures and direct identification from clinical samples is not feasible. The incubation period, medium choice, and sample preparation could differ and have an impact on the outcomes [ 91 ]. 

### Radiological Examination

Radiological findings of various forms of mucormycosis are shown in Table 4.

**Table 4:** Radiological findings of various forms of mucormycosis

Form of mucormycosis	Radiological finding
Rhinocerebral mucormycosis	Bone erosions
	Mucosal thickening
	Mass lesions inside the sinuses
	Orbital and intracranial invasions [ 92 ]
Pulmonary mucormycosis	Reverse halo sign
	Perivascular ground-glass lesion
	Nodules or masses [ 93 ]
Gastrointestinal mucormycosis	Thickening of the gut wall
	Bleeding in the stomach
	Localized or multifocal loss of bowel wall and disappearing bowel [ 94 ].

### Treatment

Zygomycetes include a diverse group of fungi with variable susceptibilities to antifungals [ 95 ]. Polyene antifungals, triazole agents, and various combination therapies are administered for the management of mucormycosis (Table 5).

**Table 5:** Medical management of mucormycosis*

	Regimens [ 96 - 122 ]	Dosage [ 96 - 122 ]	Duration of usage [ 96 - 122 ]	Management [ 96 - 122 ]	Survival rate [ 96 - 122 ]
	Amphotericin B deoxycholate	1.0-1.5 mg/kg/day	3-6 weeks	First-line drug of choice for all invasive mucormycosis (all amphotericin have equal efficacy while liposomal is least nephrotoxic)	39%
	Injection of amphotericin B lipid complex	5-10 mg/kg/day	3-6 weeks	First-line drug of choice for all invasive mucormycosis (all amphotericin have equal efficacy while liposomal is least nephrotoxic) And commonly used in pulmonary mucormycosis	71%
**First-line therapy**	Liposomal amphotericin B	5-10 mg/kg/day	3-6 weeks	First-line drug of choice for all invasive mucormycosis (all amphotericin have equal efficacy while liposomal is least nephrotoxic) and commonly used in CNS mucormycosis	67%
**Salvage therapy**	Posaconazole	300 mg twice a day 1 and 300 mg daily	3-6 months	All forms of invasive mucormycosis	60-70%
	ISAV	200 mg thrice a day and 200 mg daily (IV/oral)	102 days	Primary mucormycosis	40%
			33 days	Refractory mucormycosis	
			85 days	Intolerance to other antifungal therapy	
**Combination therapy**	Amphotericin+echinocandins (caspofungin)	Amp B (1 mg/kg/day)+caspofungin (70 mg on the first day followed by 50 mg/day)	1 week-6 months	Pulmonary mucormycosis [ 102 - 104 ], ROCM [ 105 - 109 ], disseminated [ 104 ] gastrointestinal mucormycosis [ 110 ]	54%
	Amphotericin + echinocandins (micafungin)	Not reported	4 weeks	Gastrointestinal mucormycosis [ 111 ]	20%
	Amphotericin + triazoles (posaconazole)	Not reported	1-6 months	ROCM [ 112 - 115 ], gastrointestinal mucormycosis [ 116 - 118 ], disseminated mucormycosis [ 119 ]	40-80%
	Amphotericin + triazoles (ISAV)	LAmB (10 mg/kg, given intravenously+ISAV (56 mg/kg, by oral gavage TID)	4 days	Gastrointestinal mucormycosis [ 101 ]	80%
	Amphotericin + DFX	LAmB (15 mg/kg/day)+ DFX (10 mg/kg/day)	4 days	Mucormycosis (CNS involvement)	40%
			7 days		
	Posaconazole + DFX	Posaconazole (10 mg/kg/day)+DFX (10 mg/kg/day)	Not reported	Pulmonary mucormycosis [ 99 ]	40%
**Triple therapy**	Polyene +echinocandin (caspofungin)+triazole (ISAV)	Not reported	Not reported	Pulmonary mucormycosis [ 121 ]	40-60%
	Polyene +echinocandin (caspofungin)+triazole (posaconazole)	Not reported	Not reported	Disseminated mucormycosis [ 122 ]	

*All the regimens are combined with surgical debridement wherever necessary for better outcomes. *Salvage therapy: therapy after primary therapy, often owing to the perception that the primary therapy is ineffective or the patient is intolerant.

CNS: Central nervous system, IV: Intravenous, Amp B: Amphotericin B, ROCM: Rhino-orbito-cerebral mucormycosis, ISAV: Isavuconazole, DFX: Deferasirox, LAmB: Liposomal Amphotericin B, TID: Three times a day

### Resistance during the COVID-19 pandemic

Amphotericin B is the recommended treatment for mucormycosis since it has significant *in vitro* action and a low minimum inhibitory concentration (MIC) value. The COVID-19 pandemic caused a drug crisis, after which azoles, like posaconazole, which have good *in-vitro* activity, were employed as the treatment agent [ 96 , 123 ]. Due to the higher usage of posaconazole, most strains developed resistance against it. 

Amphotericin B showed good *in-vitro* activity despite its limited use during the pandemic and can still be used. It is a good treatment option for the management of resistant infections in the future and can be considered for newer azoles, like isavuconazole as it has good *in-vitro* activity and a low mean MIC. Combination therapy may be used when monotherapy has proven ineffective; however, additional research on the interactions between various drugs is necessary to improve patient care [ 4 , 97 , 124 - 126 ]. The most effective approach for treating mucormycosis is found to be a combination of antifungal medication and surgery.

### Intrinsic resistance in mucorales

Mucorales are intrinsically resistant to azoles and other common antifungal drugs. Members of the ABC transporter superfamily may be in control of the drug efflux that leads to azole resistance. Drug resistance is most closely related to a subtype of ABC transporters called pleiotropic drug resistance (PDR) transporters. The development of azole resistance in the fungi that cause mucormycosis is poorly understood. Various azoles, such as fluconazole and VRC, can affect how sensitive these fungi are to certain amino acid alterations in one of the Cyp51 enzymes [ 127 ]. 

Eight potential PDR-type transporters are encoded in the genome of *Mucor circinelloides*. The majority of Mucorales spp. are resistant to fluconazole and VRC, whereas isavuconazole and itraconazole have been shown to have species-specific *in vitro* actions [ 10 ]. The mutants became more sensitive to posaconazole and isavuconazole when pdr1 was deleted. Deletion of *pdr2* had no impact on the susceptibility to isavuconazole despite the fact that it was discovered to be elevated by this drug [ 128 ].

## Conclusion

Early identification has become essential due to the significant rise in mortality and morbidity rate of mucormycosis during the pandemic. Due to the deadly nature of zygomycetes, it is crucial to develop a new molecular technique for its early diagnosis. Standardization of a conventional PCR for the detection of zygomycetes directly from patient samples would help in the early diagnosis and can also be performed in a resource-limited laboratory setting. 

This will also help to overcome the long time required for diagnosis, including the time needed for the culture to grow and the performance of further tests. Hence, the early identification of zygomycetes directly from samples will not only aid in faster diagnosis but also help in the right choice of antifungals. As the antifungals used against Mucorales are slowly developing resistance, further studies on the synergistic effects of drugs can be studied to administer combination therapy rather than monotherapy. 

## Acknowledgments

The authors would like to thank the management of Sri Ramachandra Institute of Higher Education and Research, Chennai, India for the provision of excellent infrastructure and facilities for research.

## Authors’ contribution

M. K. contributed to the literature search and draft preparation. P. T., S. P., and A.J. K. contributed to the conceptualization, manuscript editing, and critical review. All authors read and approved the final manuscript.

## Conflicts of interest

None.

## Financial disclosure

Self-funding.
